# 5HT3 Antagonists versus Dexamethasone in the Prevention of PONV in Patients Undergoing Laparoscopic Cholecystectomy: A Meta-Analysis of RCTs

**DOI:** 10.1155/2016/8603409

**Published:** 2016-11-06

**Authors:** Chengmao Zhou, Yu Zhu, Zhen Liu, Lin Ruan

**Affiliations:** ^1^Department of Anesthesiology, Affiliated Tumor Hospital of Guangxi Medical University, Nanning 530021, China; ^2^Zhaoqing Medical College, Zhaoqing 526000, China

## Abstract

*Background*. 5HT3 antagonist, an antiemetic alternative to dexamethasone, is an effective drug for the prevention of postoperative nausea and vomiting (PONV).* Methods*. PubMed and The Cochrane Library (from inception to June 2016) were searched for relevant RCTs (randomized controlled trials).* Results*. Seven trials, totaling 682 patients, were included in this meta-analysis. This meta-analysis demonstrated that 5HT3 antagonist was as effective as dexamethasone in preventing PONV (RR, 1.12; 95% CI, [0.86, 1.45]; *P* = 0.40) within 24 hours of laparoscopic cholecystectomy, and no significant heterogeneity was observed among the studies (*I* ^2^ = 0%; *P* = 0.98). During the early postoperative period (0–6 h), 5HT3 antagonists were superior to dexamethasone in reducing POV (RR, 0.31; 95% CI, [0.11, 0.93]; *P* = 0.04), while, in other postoperative stages (6–12 h, 12–24 h, and 0–24 h), it was not more effective in the prevention of POV than dexamethasone. And no significant difference was found in the prevention of PON between 5HT3 antagonists and dexamethasone at different postoperative periods (0–6 h, 6–12 h, 12–24 h, and 0–24 h).* Conclusions.* As a result, it is advisable to encourage 5HT3 antagonists as an alternative to dexamethasone for the prevention of PONV in patients undergoing laparoscopic cholecystectomy.

## 1. Introduction

PONV, one of the most dreaded and distressing side effects in patients undergoing laparoscopic surgery, can give rise to dehydration, anxiety, wound disruption, metabolic abnormality, prolonged recovery, and other issues [1]. Moreover, the incidence of PONV was up to 53% to 72% after laparoscopic cholecystectomy [2]. In the first 24 hours after laparoscopic cholecystectomy, PONV commonly occurred [3].

5HT3 receptor antagonists, including ondansetron, ramosetron, and granisetron, play a role in antiemetic via acting against vomiting signals in the afferent pathway from the stomach or small intestine and nucleus of the solitary tract (NTS). And it is effective in preventing PONV. As a corticosteroid, dexamethasone was first considered as an effective antiemetic drug in patients undergoing cancer chemotherapy in 1981 [4]. The idea that dexamethasone is most effective when administered at the induction rather than the termination of anesthesia had been proved by Wang et al. [5]. However, there are also quite a few side effects of dexamethasone, affecting the efficacy of PONV.

At present, whether 5HT3 receptor antagonists are effective alternatives to dexamethasone in the prevention of PONV in patients undergoing laparoscopic cholecystectomy has not been confirmed. Therefore, it is of great necessity to perform a meta-analysis to evaluate the results of published studies on this issue. As a result, a meta-analysis was performed to compare the efficacy of dexamethasone and that of 5HT3 receptor antagonists.

## 2. Materials and Methods

### 2.1. Inclusion and Exclusion Criteria

#### 2.1.1. Research Types

We choose RCTs that were limited to English texts.

#### 2.1.2. Study Subjects

We choose American Society of Anesthesiology (ASA) I or II adult patients that undergo laparoscopic surgery.

#### 2.1.3. Interventions

Intervention group received 5HT3 receptor antagonists, while the controlled group was given dexamethasone.

#### 2.1.4. Outcome Indicators

The primary outcome included the incidence of PONV in the first 24 hours after surgery, and the secondary outcomes included POV and PON in the postoperative period (0–6 h, 6–12 h, 12–24 h, and 0–24 h).

#### 2.1.5. Exclusion Criteria

Exclusion criteria include repeated studies and studies with incomplete data.

### 2.2. Search Strategy

PubMed, Embase, The Cochrane Library, and CNKI were searched (from inception to June 2016) for RCTs on efficacy and safety of dexamethasone and 5HT3 receptor antagonists in preventing PONV. The following search terms were included: “ondansetron”, “ramosetron”, “palonosetron”, “tropisetron”, “granisetron”, “5-HT3 receptor antagonists”, “nausea”, “vomiting”, “dexamethasone” and “laparoscopic cholecystectomy”.

### 2.3. Literatures Screening and Data Extraction

According to inclusion and exclusion criteria, two reviewers independently screened literatures and extracted data and then cross-checked with each other. The two discussed or consulted a third party when there was a disagreement.

### 2.4. Quality Evaluation

We evaluated methodological quality of included studies based on risk of bias of the Cochrane Handbook for Systematic Reviews of Interventions (Version, 5.1.0) [2] and then adopted modified Jadad scale to assess the quality. The primary categories consisted of (1) randomization; (2) description of withdrawals and drop-outs; (3) blinding (personnel and participants); (4) incomplete outcome data, whether described withdrawals or drop-outs.

### 2.5. Statistical Analysis

We performed the meta-analysis by adopting RevMan 5.2. Enumeration data were expressed as relative risk (RR) with 95% CI, and measurement data were represented through weighted mean difference (WMD) with 95% CI. A heterogeneity test was done on included studies via *χ* ^2^ test, and when *α* = 0.05 and *P* ≤ 0.05, heterogeneity was considered present. Furthermore, a quantitative analysis was conducted on heterogeneity by adopting *I* ^2^ value, and heterogeneity existed when *I* ^2^ ≥ 50%. We adopted a fixed-effects model to do a meta-analysis when there was no heterogeneity. A random-effects model was employed when each study showed statistical heterogeneity rather than clinical heterogeneity or when the differences had no significance. And a descriptive analysis approach was used when the heterogeneity was too large.

## 3. Results

### 3.1. Identification of Eligible Studies

We identified a total of 74 potentially relevant abstracts. Only 8 of them matched with the inclusion criteria after the abstracts have been reviewed. One of them, without complete data, was excluded [12].

**Figure 1:**
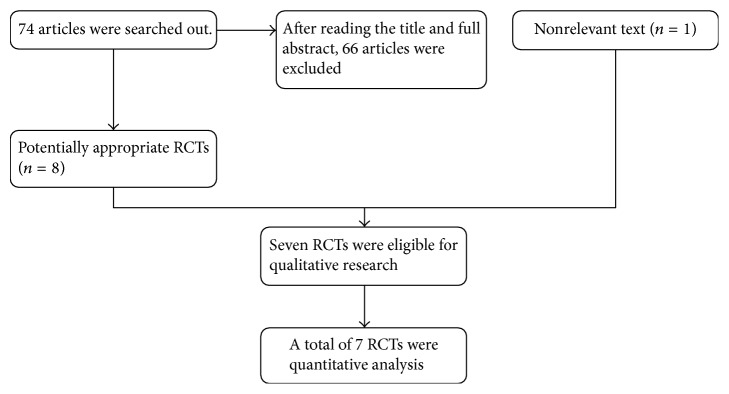
Flow diagram.

At last, 7 studies [6–11, 13] were enrolled in this meta-analysis.  Figure 1 presents search strategy and study selection.

### 3.2. Study Characteristics

The characteristics of all included studies are shown in Table 1. And Jadad scale results are also shown in Table 1.

## 4. Primary Outcomes of Meta-Analyses

### 4.1. PONV (0–24 Hours)

**Figure 2:**
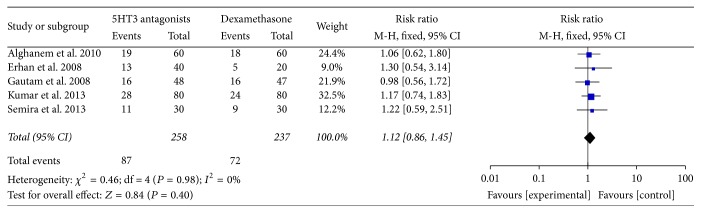
PONV (0–24 hours).

The results are presented in Figure 2. We studied PONV within 24 hours in five trials. Compared with dexamethasone, 5HT3 receptor antagonists were not related to a significant decreasing of incidence of PONV (RR, 1.12; 95% CI, [0.86, 1.45]; *P* = 0.40), but no significant heterogeneity was found among the remaining trials (*I* ^2^ = 0%; *P* = 0.98).

### 4.2. Postoperative Nausea at Different Stages

**Figure 3:**
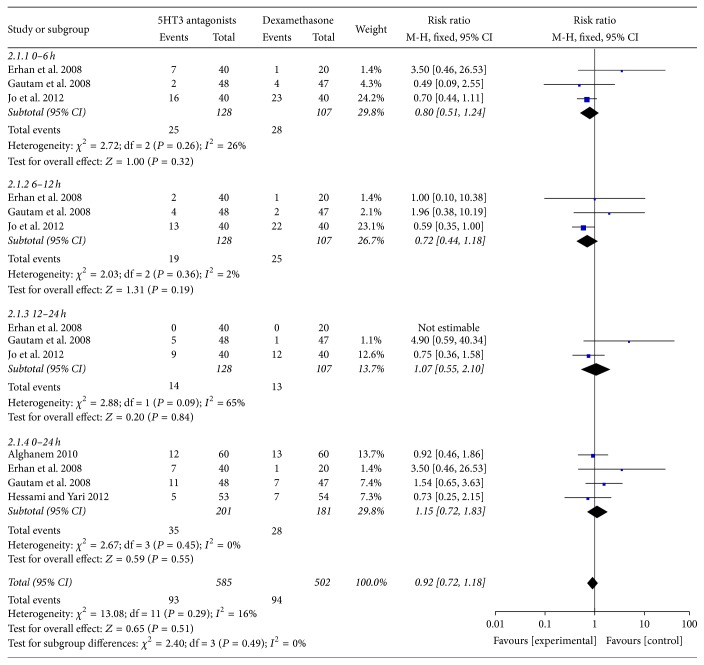
Postoperative nausea at different stages.

On the basis of the postoperative stage, we also did a subgroup meta-analysis to analyze the efficacy of 5HT3 receptor antagonists in the prevention of PONV compared with that of dexamethasone. 5HT3 receptor antagonists were not superior to dexamethasone on preventing PON (postoperative nausea) during some of the time periods in the first 24 hours after surgery: 0–6 hours (RR, 0.80; 95% CI, [0.51, 1.24]; *P* = 0.32), 6–12 hours (RR, 0.72; 95% CI, [0.44, 1.18]; *P* = 0.19), 12–24 hours (RR, 1.07; 95% CI, [0.55, 2.10]; *P* = 0.84), and 0–24 hours (RR, 1.15; 95% CI, [0.72, 1.83]; *P* = 0.55) (Figure 3). The 0% *I* ^2^ value indicated that there was no significant heterogeneity.

### 4.3. Postoperative Vomiting at Different Stages

**Figure 4:**
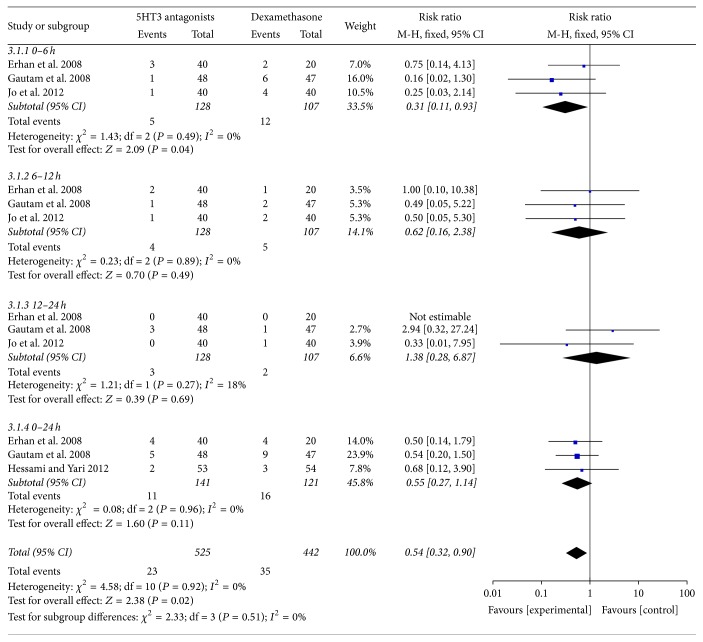
Postoperative vomiting at different stages.

No statistically significant difference was observed between the two groups during some of the time periods within 24 hours after surgery: 0–6 hours (RR, 0.31; 95% CI, [0.11, 0.93]; *P* = 0.04), 6–12 hours (RR, 0.62; 95% CI, [0.16, 2.38]; *P* = 0.49), 12–24 hours (RR, 1.38; 95% CI, [0.28, 6.87]; *P* = 0.69), and 0–24 hours (RR, 0.55; 95% CI, [0.27, 1.14]; *P* = 0.11) (Figure 4).

### 4.4. Publication Bias Analysis

We conducted a funnel plot analysis on included studies, which showed good symmetrical results, indicating that this study is less likely to be affected by publication bias.

## 5. Discussion

This is the first meta-analysis to compare the effectiveness of 5HT3 receptor antagonists with that of dexamethasone in the prevention of PONV after laparoscopic cholecystectomy. We employed a fixed-effects model to do the pooled meta-analysis of 7 RCTs, suggesting that no significant differences between 5HT3 receptor antagonists and dexamethasone were found with regard to the incidence of PONV during the first 24 hours after laparoscopic surgery.

Dexamethasone, as glucocorticoids, plays a positive role in PONV in patients undergoing chemotherapy or general anesthesia. However, its mechanism of antiemetic remains unclear. The antiemetic mechanism may be that dexamethasone inhibits production and release of 5-HT in central nerves and peripheral region, changes permeability of blood brain barrier (BBB) to 5-HT, and thus reduces 5-HT's function on concentration of intestinal chemical sensors [14]. However, dexamethasone has its adverse reactions, such as, increase of infection, inhibition of adrenal gland, and delayed wound healing.

The exact mechanism of ramosetron, granisetron, and ondansetron in the prevention of PONV is unknown, but the drugs may function through blocking 5HT3 receptors sites at area postrema and NTS.

In summary, 5HT3 receptor antagonists were as effective and safe as dexamethasone in the prevention of PONV. However, there are some limitations of this meta-analysis. First, no gold standard for the definition of PONV was provided. Furthermore, this meta-analysis was performed on the basis of studies published in English language, which may inflict bias. Moreover, the sample sizes of the studied trials were small or moderate. Moreover, no difference between the two groups was observed in the incidence of PONV (0–24 h) because of the small sample size and lacking evidence. Therefore, caution should be put on our findings, and larger studies comparing 5HT3 receptor antagonists with dexamethasone are needed to support our finding.

## Figures and Tables

**Table 1:** Characteristics and Jadad scores of the included studies in the meta-analysis.

Author (publication year)	Country	Headcount	Grouping	Jadad score	Randomized method	Concealment allocation	Blinding	Follow-up
Erhan et al. 2008 [6]	Turkey	80	Ondansetron Granisetron Dexamethasone 0.9% NaCl	5	1	1	2	1

Hessami et al. 2012 [7]	Iran	104	Granisetron Dexamethasone Placebo	5	2	1	1	1

Semira et al. 2013 [8]	India	100	Ondansetron Dexamethasone Saline Gabapentin	6	2	1	2	1

Alghanem et al. 2010 [9]	Jordan	180	Ondansetron Dexamethasone 0.9% NaCl	5	1	1	2	1

Gautam et al. 2008 [10]	Nepal	155	Ondansetron Dexamethasone Ondansetron + dexamethasone	6	2	1	2	1

Kumar 2013 [13]	India	320	Ondansetron Dexamethasone Ondansetron + dexamethasone Placebo	4	1	1	1	1

Jo et al. 2012 [11]	Korea	120	Ramosetron Dexamethasone Ramosetron + dexamethasone	6	2	1	2	1
